# Pragmatic Person-Centered Care for Diabetes in Myanmar: A Narrative Review and Expert Insights From an Advisory Board Meeting

**DOI:** 10.7759/cureus.116808

**Published:** 2026-09-23

**Authors:** Tint Swe Latt, Than T Aye, Ko Ko, Khin Saw Than, Sanjay Kalra, Nitin Kapoor, Thein Myint, San S Win, Htet Htet Khin, Ni Ni Hlaing, Kyaw Thu Yaa, Maung Maung Thant, Kyar Nyo S Myint, Yar Pyae

**Affiliations:** 1 Endocrinology, Myanmar Diabetes Association, Yangon, MMR; 2 Diabetes and Endocrinology, Grand Hantha International Hospital, University of Medicine 2, Yangon, Yangon, MMR; 3 Endocrinology, University of Medicine 2, Yangon, Yangon, MMR; 4 Endocrinology, Grand Hantha International Hospital, Yangon, MMR; 5 Endocrinology, Bharti Hospital, Karnal, IND; 6 Endocrinology, Diabetes and Metabolism, Christian Medical College and Hospital, Vellore, Vellore, IND; 7 Medicine, University of Medicine 1, Yangon, Yangon, MMR; 8 Medical Unit 1, Yangon General Hospital, Yangon, MMR; 9 Diabetes and Endocrinology, Yangon General Hospital, University of Medicine, Mandalay, Mandalay, MMR; 10 Diabetes and Endocrinology, University of Medicine, Mandalay, Mandalay, MMR; 11 Endocrinology, University of Medicine, Magway, Magway, MMR; 12 Endocrinology and Medicine, Thingangyun General Hospital, University of Medicine 2, Yangon, MMR; 13 Diabetes and Endocrinology, North Oakkalapa General Hospital, University of Medicine 2, Yangon, MMR

**Keywords:** diabetes mellitus, disease management, holistic health, myanmar, patient-centered care

## Abstract

Diabetes causes considerable morbidity and mortality in Myanmar. A pragmatic person-centered care (PCC) approach offers a holistic and individualized strategy. This expert opinion paper explores perspectives from Myanmar and Indian experts on diabetes burden, the role of PCC, and the barriers to its implementation. An advisory board meeting in December 2024 included expert-led sessions. Patient, physician, healthcare system, and curriculum-related challenges in implementing PCC were identified. However, Myanmar's cultural strengths deeply rooted in Buddhist philosophy may support PCC adoption. Personalized pharmacotherapy was emphasized. Metformin remained a preferred initial agent, while sodium-glucose cotransporter-2 inhibitors and glucagon-like peptide-1 receptor agonists were favored for individuals with high cardio-renal risk, independent of metformin use.

## Introduction and background

Diabetes is a significant public health concern with its burden steadily rising across the world [1-3]. The scenario is no different in the Asian country of Myanmar, where the International Diabetes Federation Diabetes Atlas reported 2.34 million adults living with diabetes in 2021, with the number projected to rise to 3.43 million by 2045 [4]. Despite the presence of established conventional approaches, people with diabetes (PwD) often face significant challenges contributing to suboptimal glycemic control [5]. A pragmatic person-centered care (PCC) approach serves as a framework to overcome the challenges of the conventional approach [6].

PCC is a holistic approach that empowers patients for effective self-management by aligning care with individual preferences, improving self-care, glycemic control, and medication adherence [5]. The European Association for the Studies on Diabetes and the American Diabetes Association (ADA) consensus report outlines prevention of diabetic complications and optimization of quality of life (QoL) as key goals of PCC, achievable through 13 care principles focused on individualizing treatment based on patient characteristics and preferences [7]. Healthcare disparities remain a concerning issue in resource-limited countries [8]. Models such as PCC, which focus on optimizing resource use, promoting patient rights, and ensuring high-quality healthcare services, can offer potential solutions to address these challenges [9]. By integrating a narrative review of the current literature with expert insights generated from an advisory board meeting, this paper aims to examine the burden of diabetes, the role of PCC in the management of diabetes, and the barriers to and enablers of its implementation in the specific context of Myanmar.

## Review

An advisory board meeting was conducted on 8 December 2024 in Myanmar. A comprehensive literature search was performed to develop a discussion guide for the meeting, comprising sessions on PCC-related topics by expert speakers, followed by group discussions and opinions from the experts. The targeted, non-systematic literature search was conducted using electronic databases, including PubMed and Google Scholar, for relevant articles published up to December 2024, using search terms such as "person-centered care", "patient-centered care", "diabetes mellitus", "type 2 diabetes", "Myanmar", "diabetes self-management", "pharmacotherapy", and "stewardship". English-language clinical practice guidelines, consensus statements, systematic reviews, meta-analyses, observational studies, and other pertinent publications relevant to the discussion topics were screened and selected by the authors based on their relevance, recency, and applicability to the Myanmar context. The search was intended to provide a focused evidence base for the key discussion topics and to inform the development of the recommendations. As this was a targeted, non-systematic literature search for a narrative review, no formal risk-of-bias assessment, quantitative evidence synthesis, or meta-analysis was performed. The meeting was initiated with a quiz aimed at assessing the perspectives of the experts on PCC. This was followed by a discussion on the topics: diabetes, defining PCC, barriers to PCC, Myanmar's strengths in the fight against diabetes, PCC stewardship, motivational therapeutics, and pharmacotherapy for diabetes management. A moderator steered the discussions. A total of 39 experts from Myanmar and India participated in the advisory board meeting. Experts were selected based on their demonstrated experience in diabetes care and management, active involvement in the care of people with diabetes, and contributions to clinical practice, research, medical education, or professional societies related to diabetes. The advisory board discussions were documented through detailed note-taking by a designated rapporteur. Expert opinions expressed during the group discussions were collated and synthesized narratively by the moderator and captured the key perspectives, considerations, and areas of agreement or differing views raised during the discussions. No formal voting, predefined consensus thresholds, or structured thematic analysis were applied. This paper represents a narrative review integrated with expert opinion. The synthesized expert inputs were then incorporated into the discussion and expert opinion sections of the paper. Table 1 presents the discussion guide for the advisory board meeting.

**Table 1:** Discussion guide for the advisory board meeting ADA: American Diabetes Association; OAD: Oral antidiabetic drug; PCC: Person-centered care; PCOS: Polycystic ovary syndrome

Topics	Subtopics covered
Quiz	Questions on assessing obesity in a patient-centered way, guideline-based management of obesity and PCOS in people with diabetes, pillar of PCC, diet prescription, barriers to PCC, alternative medication approach
Well-being and diabetes	Impact of diabetes on physical, mental, social, and emotional well-being, recommendations by the ADA for diabetes management, behavioral therapy
PCC	Challenges in the management of diabetes, description and definition of PCC, atreya quadruple and quintessential quincunx models, categorization of patients based on phenotype and sapiotype
Barriers to PCC	Person-related barriers, physician-related barriers, healthcare system-related barriers, curriculum-related barriers
Myanmar’s strengths in the fight against diabetes	Strength of family, strength of community, strength of religion and spirituality, strength of traditional Myanmar wisdom in achieving 50% PCC
PCC stewardship	Types of stewardship, aspects to be considered while following the stewardship
Lifestyle modifications and pharmacotherapy	Choice of OAD, choice of antihypertensives, choice of statins, choice of body weight management
Motivational therapeutics	Approach towards motivational therapeutics, tools of motivational therapy

Results

Insights From the Advisory Board Meeting

Based on the responses to the quiz questions in Figure 1, which served as an informal, non-validated tool to assess the experts' baseline understanding of and perspectives on PCC, the responses indicated familiarity with and favorable perspectives toward the holistic PCC approach to diabetes management. The responses also reflected recognition of patient characteristics and physician-related barriers to implementing PCC in clinical practice, as well as the importance of prioritizing patients' needs and preferences through shared decision-making.

**Figure 1:**
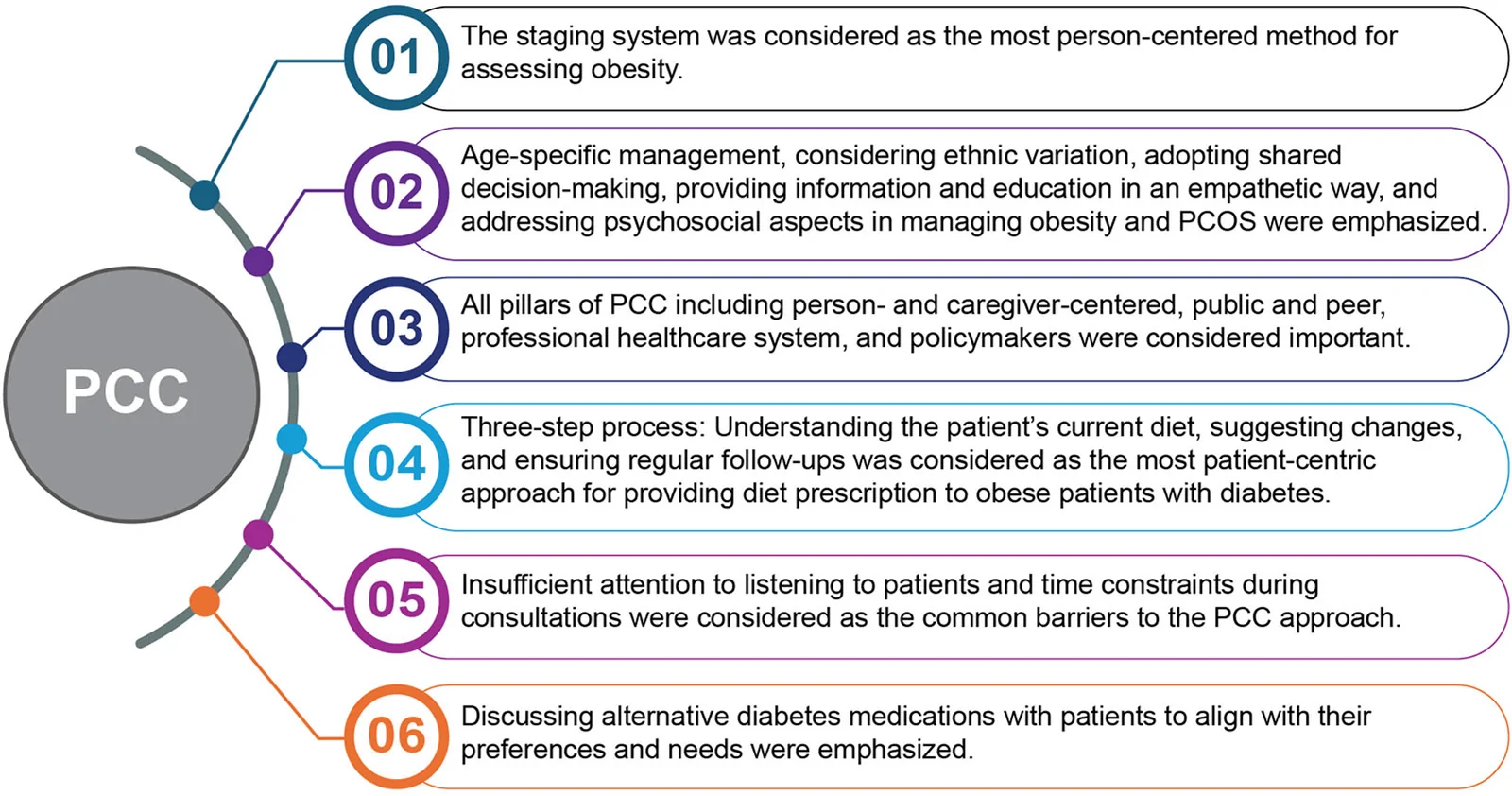
Responses reflecting the understanding of the experts regarding the PCC approach PCC: Person-centered care; PCOS: Polycystic ovary syndrome Figure conceptualized and created by the authors using Microsoft PowerPoint and Microsoft Word (Microsoft® Corp., Redmond, WA); the final artwork was professionally enhanced for publication.

Diabetes and Well-Being

According to the Global Burden of Disease (GBD) 2021, diabetes accounted for 79.2 million disability-adjusted life years (DALYs), with type 2 diabetes mellitus (T2DM) accounting for 95.4% of this burden [10]. Similarly, the situation in Myanmar is concerning, reflecting the typical iceberg phenomenon of disease. An estimated 7.1% of the population aged 20-79 years is affected by diabetes, representing only the visible tip of the iceberg, while 51.7% of those with diabetes remain undiagnosed [4]. Diabetes, a chronic lifelong condition, often recurs and worsens over time, impacting both physical and mental health [11]. PwD are at increased risk of anxiety, maladjustment, depression, and mental disorders (in severe cases), which impede treatment adherence and self-management, while contributing to organ damage and elevating the risk of complications [11,12].

A multinational study reported that the dynamic and chronic nature of diabetes, and the constant need for PwD to adjust their diet and medication, was the biggest disabler in managing diabetes. The financial burden of laboratory investigations and treatment was identified as a significant barrier to self-care [13]. In Myanmar, the total diabetes-related health expenditure in 2021 was estimated at 457.3 million USD (914.6 billion MMK), projected to increase to 554.3 million USD (1.1086 trillion MMK) by 2045 [4]. The study also highlighted work-related travel, discrepancy between patient and healthcare professional (HCP) perceptions of care, including conflicting self-management advice, as key challenges in managing the condition [13].

Diabetes self-management education and support (DSMES) is a critical component of the treatment plan, as important as pharmacotherapy selection. It is customized to the individual's context, including their beliefs and preferences [7]. Evidence shows that DSMES is effective in improving glycemic control and patient-related outcomes, particularly when integrated with psychosocial interventions [14,15]. The opinions of the experts on the diabetes burden and well-being are aligned well with the existing literature and detailed below.

Expert opinions: The experts emphasized that diabetes affects overall well-being beyond glycemic control, influencing the physical, emotional, social, occupational, and sexual aspects of life. A large proportion of PwD experience stress, anxiety, depression, frustration, and diabetes burnout, which can negatively impact self-management and QoL. The panel highlighted that diabetes-related challenges span biomedical, psychological, social, and environmental domains, necessitating a holistic approach to care. They recommended integrating psychological support, including counseling, cognitive behavioral therapy, mindfulness-based interventions, and resilience-building programs, alongside routine diabetes management. Consistent with ADA 2024 Standards of Care, the experts stressed the importance of incorporating DSMES at diagnosis, annually, and during life transitions or complications. They also highlighted the role of regular physical activity, walking, and adequate sleep in improving glycemic control and overall health, advocating for a multidisciplinary approach to enhance the well-being and QoL of PwD.

PCC Approach in the Management of Diabetes

In Myanmar, several sociocultural practices, such as attributing diabetes to "germs", excessive physical activity, or past misdeeds, often influence treatment choices and reliance on traditional medications [16]. This highlights the importance of implementing tailored, culturally sensitive interventions to ensure timely, individualized care, and address barriers influenced by cultural beliefs, social circumstances, and other determinants of health [7,17].

PCC is characterized by a holistic approach that incorporates an individual's comorbidities and prognoses; prioritizes respect for their preferences, needs, and values; and ensures that clinical decisions are guided by these values [18]. The ADA 2025 standards of care embrace this framework, emphasizing the individualization of diabetes care and acknowledging PwD as unique individuals. This approach considers patients' values, preferences, social determinants of health, barriers to care, comorbid conditions, degree of hyperglycemia, susceptibility to medication side effects, and risk of complications, and emphasizes effectively communicating the balance of risks and benefits of each intervention [18]. In Myanmar, a diabetes care model was developed to bridge rural-urban disparities by providing accessible, comprehensive, and efficient care in both urban and rural communities across all healthcare levels. It focused on community mobilization, empowering healthcare staff, involving communities in care planning, applying the universal health coverage concept, managing World Health Organization (WHO)-recommended essential drugs, and preventing other non-communicable diseases [19].

Understanding patient phenotypes is also important in managing diabetes. Categorizing patients into distinct phenotypes enables tailored therapies and appropriate follow-up, as well as treatment intensity based on the patient's phenotype and disease stage. It also aids in understanding underlying dysglycemia, assessing risk of complications, and targeting therapies more effectively. Severe insulin-deficient diabetes, severe insulin-resistant diabetes, mild obesity-related diabetes, and mild age-related diabetes are the four phenotypes of PwD reported in the literature [20]. The opinions of the experts on the PCC approach in the management of diabetes are in line with the literature and are detailed below.

Expert opinions: The experts emphasized that PCC is fundamental to effective diabetes management and should be tailored to the individual needs, preferences, values, and circumstances of each PwD. They noted that, while international guidelines provide a useful framework, their implementation may be influenced by local cultural beliefs, healthcare barriers, and socioeconomic factors. The experts highlighted the importance of compassionate, respectful, and nonjudgmental communication, with counseling focused on understanding patients’ challenges, treatment preferences, convenience, comfort, and affordability. Various frameworks, including the Atreya quadruple and the quintessential quincunx model, were discussed as approaches to support comprehensive, team-based diabetes care. The experts further stressed that management strategies should be individualized based on factors such as metabolic phenotype, health literacy, numeracy, and readiness to engage in self-care, thereby facilitating informed therapeutic decisions and optimizing consultation time and patient outcomes.

Barriers to the PCC Approach

Despite guidelines emphasizing the PCC approach for diabetes management, resource-limited countries face several barriers to its implementation at patient, HCP, and healthcare system levels. Many PwD have misconceptions regarding diabetes, demonstrate a lack of self-motivation for self-care, struggle to adopt dietary modifications, and present with a low literacy level, inhibiting them from reading, writing, and understanding health information. Additionally, the cost of laboratory tests and diabetic drugs has been reported as a barrier to PCC implementation [21].

A key HCP-level barrier to PCC in diabetes care is limited collaboration and lack of specialized training, resulting in an insufficient understanding of the latest management practices, reliance on outdated information, and limited capacity to effectively teach and motivate patients. Effective communication, a cornerstone of PCC, also remains a challenge due to HCPs' deficiencies in basic communication and counseling skills. Additionally, limited knowledge regarding socio-culturally tailored diabetes diets impedes the provision of personalized care [21]. However, a study evaluating the impact of collaborative primary care comprising both HCPs and patients highlighted that patients were satisfied with the valuable information provided by HCPs regarding diabetes care [22].

The healthcare systems in resource-limited countries often lack well-integrated diabetes services. In one study, patients underscored the unavailability of experts to provide timely care, leading to frequent interruptions in laboratory services and an unsustainable supply of essential diabetes medications [21]. High patient loads further limit the time allocated for HCP consultations, leading to insufficient patient education, counseling, and the ability of HCPs to establish trustworthy partnerships with their patients [21,22]. The experts in the present study also expressed the person-, physician-, and healthcare system-related barriers to implementing the PCC approach. Furthermore, they highlighted the curriculum-related barriers that prioritize a disease-specific approach over a PCC-focused approach, which are detailed below and in Table 2.

**Table 2:** Expert opinions on knowledge, attitude, and practice barriers in implementing person-centered care (PCC) for diabetes care in Myanmar Original table developed by the authors based on expert opinions generated during the advisory board discussion.

Knowledge barriers	Attitude barriers	Practice barriers
Person-related barriers come from the knowledge, attitudes, and practices of individual patient.	Patients may resist adopting new behaviors like smoking cessation, reducing alcohol, or exercising regularly.	Patients may lack confidence in their knowledge, relying solely on healthcare providers, leading to minimal participation in care decisions.
Patients may lack understanding of their illness, treatment options, and medical terminology, leading to misconceptions, poor informed decision-making, and reduced treatment adherence.	Belief that health outcomes are predetermined can lead to a lack of motivation for active participation in care.	Competing priorities (e.g., work, family) can result in missed appointments, inconsistent follow-ups, and poor treatment adherence.
Incorrect knowledge from unreliable sources (e.g., myths about medications like metformin causing renal failure) can result in inappropriate disease management.	Excessive fear or anxiety may result in avoidance behaviors, reducing engagement in necessary care.	Difficulty in tasks like blood glucose monitoring or medication adherence can hinder effective disease control.
Patients may be unaware of available support services, educational materials, and community programs that could aid in managing their condition.	Negative past experiences can lead to skepticism, hindering patient-provider relationships and care engagement.	Adopting and sustaining healthy habits (e.g., diet, exercise) is difficult due to lack of support, resources, or time constraints.
Managing chronic conditions like long-standing diabetes with multiple complications requires advanced health knowledge. Limited understanding hinders self-monitoring, self-management, and quality of care.	Patients may feel incapable of managing their illness, believing their efforts won’t impact health outcomes.	Misalignment between patient concerns and provider recommendations can reduce comfort and engagement in care.
-	Cultural beliefs about health, illness, and treatment can affect adherence, lifestyle changes, and overall participation in care.	High healthcare costs can limit access to essential medications and treatments, especially in out-of-pocket payment systems.

Expert opinions: The experts identified several physician-related, healthcare system-related, and curriculum-related barriers to the implementation of PCC. Physician-related barriers included limited consultation time, inadequate awareness and understanding of PCC principles, and attitudes that may hinder empathetic and effective communication with PwD. Healthcare system-related barriers comprised limited availability of specialized diabetes services, disparities between urban and rural healthcare settings, high treatment costs, regulatory constraints, medicolegal concerns, and inadequate coordination among healthcare providers, resulting in fragmented care delivery. Curriculum-related barriers included a predominant focus on disease-specific management and standardized treatment protocols, with insufficient emphasis on PCC, communication skills, psychosocial care, cultural competency, and collaborative practice. The experts also highlighted limited opportunities for continuing professional education, as well as time and resource constraints, as important challenges to the wider adoption of PCC in clinical practice.

Myanmar's Strengths in the Fight Against Diabetes

PwD are often nested within family and social environments, which significantly influence their diabetes care [23,24]. Family members provide instrumental support (scheduling follow-up appointments, timely insulin administration), and emotional support (alleviating diabetes-related distress, offering encouragement) [23]. A systematic review reported that DSME integrated with family support effectively reduced glycated hemoglobin (HbA1c) levels by 1% and improved perceived support, medication adherence, psychosocial aspects, self-efficacy, diabetes complications, overall QoL, and proved cost-effective [23]. Correspondingly, an umbrella review highlighted that shared family goals of maintaining fitness can positively support PwD in their diabetes management journey. Family members can contribute by engaging in physical activities together, adhering to a healthy diet, ensuring timely meals, and assisting in coordinating medication schedules [24]. In Myanmar, where family plays a central caregiving role, a study found a significant association between family support and medication adherence in PwD, highlighting its positive influence on treatment adherence [25].

In terms of religion, Buddhist practices significantly influence daily life in Myanmar and can support diabetes management when integrated with modern medicine [26]. Buddhist principles encourage PwD to pursue correct knowledge, avoid myths, and seek appropriate guidance for their condition. Additionally, it promotes lifestyle modifications and emphasizes the value of family and community support in managing diabetes effectively [26]. The noble eightfold path of Buddhism, emphasizing discipline, meditation, and self-restraint, offers a framework that supports modern diabetes management. Mindfulness meditation can aid in reducing diabetes distress, pain, anxiety, and depression, as well as controlling hypertension and diabetes [26]. Traditional medications also play an important role in Myanmar's healthcare system, supported by the government. Evidence from the literature suggests that traditional medicines are widely used in Myanmar, including among people with diabetes, and that some individuals may prefer them as a first choice for health-seeking, citing reasons such as trust, traditional beliefs, advice from others, affordability, and skepticism toward Western medicines [27]. However, these findings are based on specific populations and sources and may not be representative of the Myanmar population as a whole.

A systematic review reported a positive relationship between improvement in diabetes conditions and spirituality and religiosity positively influenced diabetes outcomes by enhancing their ability to cope with diabetes-related stress, leading to better glycemic control [28]. Given the significance of religious practices in Myanmar, religious leaders can play a potential role in raising community awareness of diabetes management. On a similar line, Bhowmik et al. described a successful initiative in Bangladesh, where religious leaders were trained on diabetes-related issues to enhance societal awareness [29]. More recent evidence also supports the potential value of culturally tailored interventions in diabetes care, with a 2026 systematic review and meta-analysis reporting improvements in self-management behaviors and glycemic control among people with T2DM [30]. The opinions of the experts regarding the strength of family values, religious practices, and the involvement of the community in the management of diabetes are in accordance with the reported literature Figure 2.

**Figure 2:**
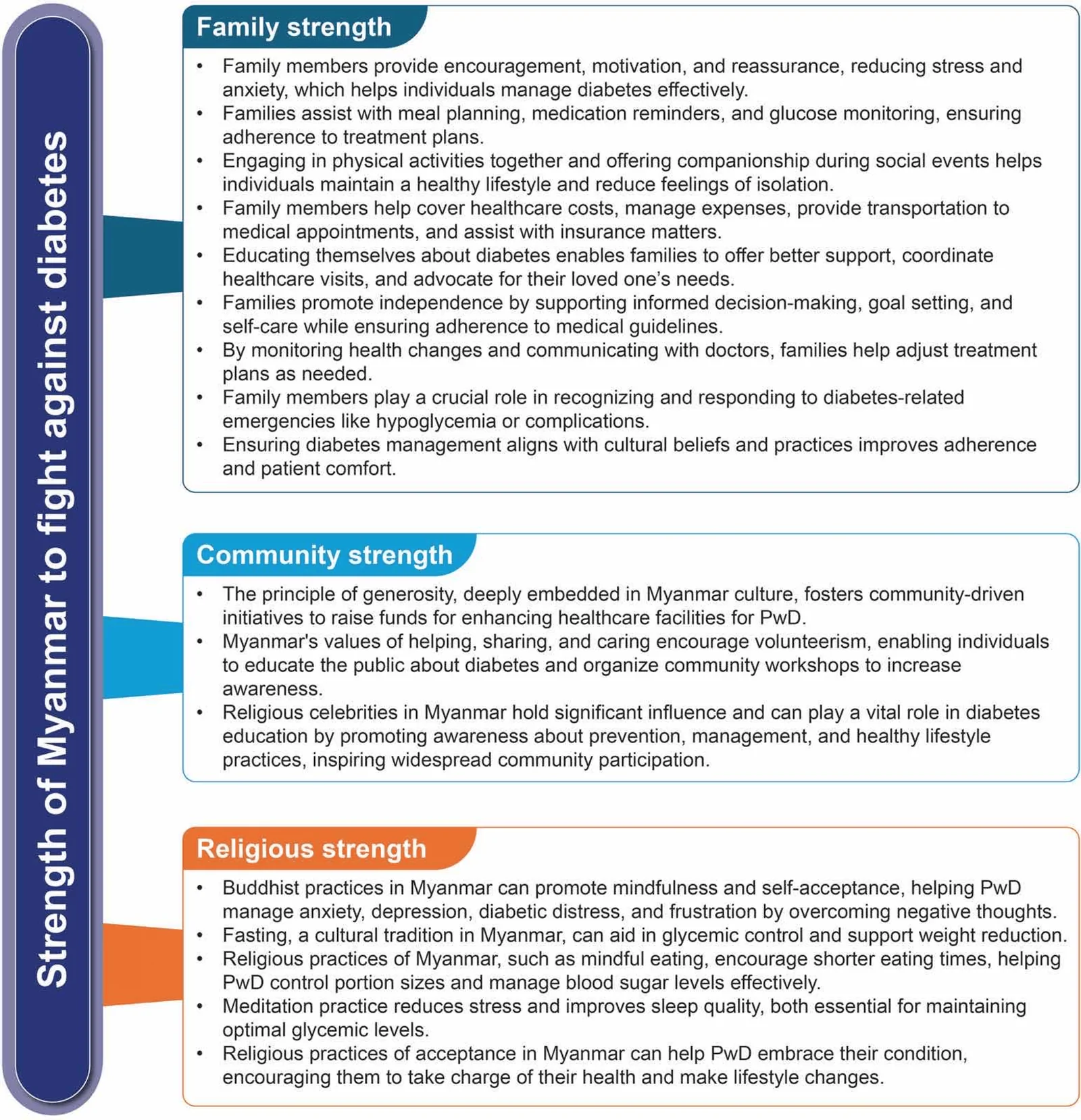
Expert opinion on Myanmar's strengths in the fight against diabetes PwD: People with diabetes Figure conceptualized and created by the authors using Microsoft PowerPoint and Microsoft Word (Microsoft® Corp., Redmond, WA); the final artwork was professionally enhanced for publication.

PCC Stewardship

The term stewardship is commonly used in relation to antimicrobial stewardship, aimed at optimizing medication use, improving patient outcomes, reducing adverse effects (AEs), and maintaining cost-effectiveness [31]. Building upon this description, the experts in the present study advocated for a similar stewardship approach in diabetes management aligned with PCC. While diabetes stewardship for PCC remains less explored, existing research primarily focuses on pharmacy and medication stewardship in diabetes management [32].

A study by Drincic et al. reported that diabetes pharmacy stewardship, along with a glucose management program, can help in controlling the glycemic level of PwD. The program emphasized optimizing medication use, accurate insulin prescribing, error prevention, and enhancing safety in inpatient care. Pharmacists played a key role in reviewing regimens and educating staff and patients [32]. Similarly, a five-year stewardship program demonstrated a 14% increase in insulin administration accuracy among nurses, a decrease in hypoglycemia from 8.27% to 5.03%, and a 15.20% increase in patient education about insulin injection [33]. Though literature specific to PCC stewardship is limited, the experts in the present study provided their opinion on having a PCC stewardship that will focus on the development of policies, processes, and practices encouraging PCC for PwD, as summarized below.

Expert opinions: The experts emphasized that stewardship is essential for the successful implementation of PCC and should encompass policies, processes, and clinical practices that support its integration into routine diabetes care. They noted that policy-level initiatives should promote the incorporation of PCC principles into healthcare education, while process-level efforts should strengthen knowledge and skills through continuous education and case-based discussions. Practice-level stewardship should focus on the consistent application of PCC in clinical settings. As part of the advisory board discussion, the experts proposed a conceptual framework describing stewardship across four interconnected domains: metabolic, macro, meso, and micro stewardship. Metabolic stewardship focuses on improving metabolic health while incorporating local cultural, traditional, and spiritual practices. Macro stewardship involves national and regional initiatives that support health promotion and public health policies. Meso stewardship operates at the institutional level, promoting PCC through healthcare systems, hospitals, and educational programs. Micro stewardship focuses on individual patient interactions, including clear communication regarding treatment goals, medication benefits, potential AEs, and appropriate medication use to enhance understanding, adherence, and clinical outcomes among people with diabetes.

Lifestyle Modifications and Pharmacological Management of Diabetes

The ADA 2025 guidelines emphasize having a PCC approach to guide the choice of antidiabetic drugs, focusing not only on achieving glycemic targets but also considering their impact on comorbidities, weight, cardiorenal outcomes, cost-effectiveness, hypoglycemia risk, potential AEs, drug accessibility, and patient tolerability [34]. For body weight management, the ADA 2025 guidelines recommend frequent counseling sessions (16 sessions over six months), emphasizing dietary modifications, increased physical activity, and behavioral strategies to create a daily 500-750 kcal deficit. If these interventions are not available, structured in-person or remote behavioral counseling programs should be considered [35]. Obesity treatment should be personalized, considering structural, cultural, and socioeconomic factors that affect nutrition and food choices, including food insecurity, access to healthy food, and social determinants of health [35]. Nevertheless, therapy choices should also account for comorbidities, side effects, cost, accessibility, and individual goals and preferences. Evidence-based, cost-effective treatment options can improve healthcare access and reduce financial burden for individuals with obesity [35]. Considering their weight management benefits, the preferred pharmacotherapy for PwD and overweight/obesity is a glucagon-like peptide-1 receptor agonist (GLP-1 RA) or a glucose-dependent insulinotropic polypeptide + GLP-1 RA with greater weight loss efficacy [35].

Antihyperglycemic drugs include oral agents (metformin, sodium-glucose cotransporter-2 inhibitors (SGLT-2is), GLP-1 RAs, dipeptidyl peptidase-4, pioglitazone, sulfonylureas) and injectable agents (GLP-1 RAs, insulin). The choice of drugs based on glycemic level and comorbidities is detailed in Figure 3 [34]. Along with medication choice, regular monitoring and dose adjustments are also crucial. Current standards recommend reevaluating diabetes medications every three to six months [34].

**Figure 3:**
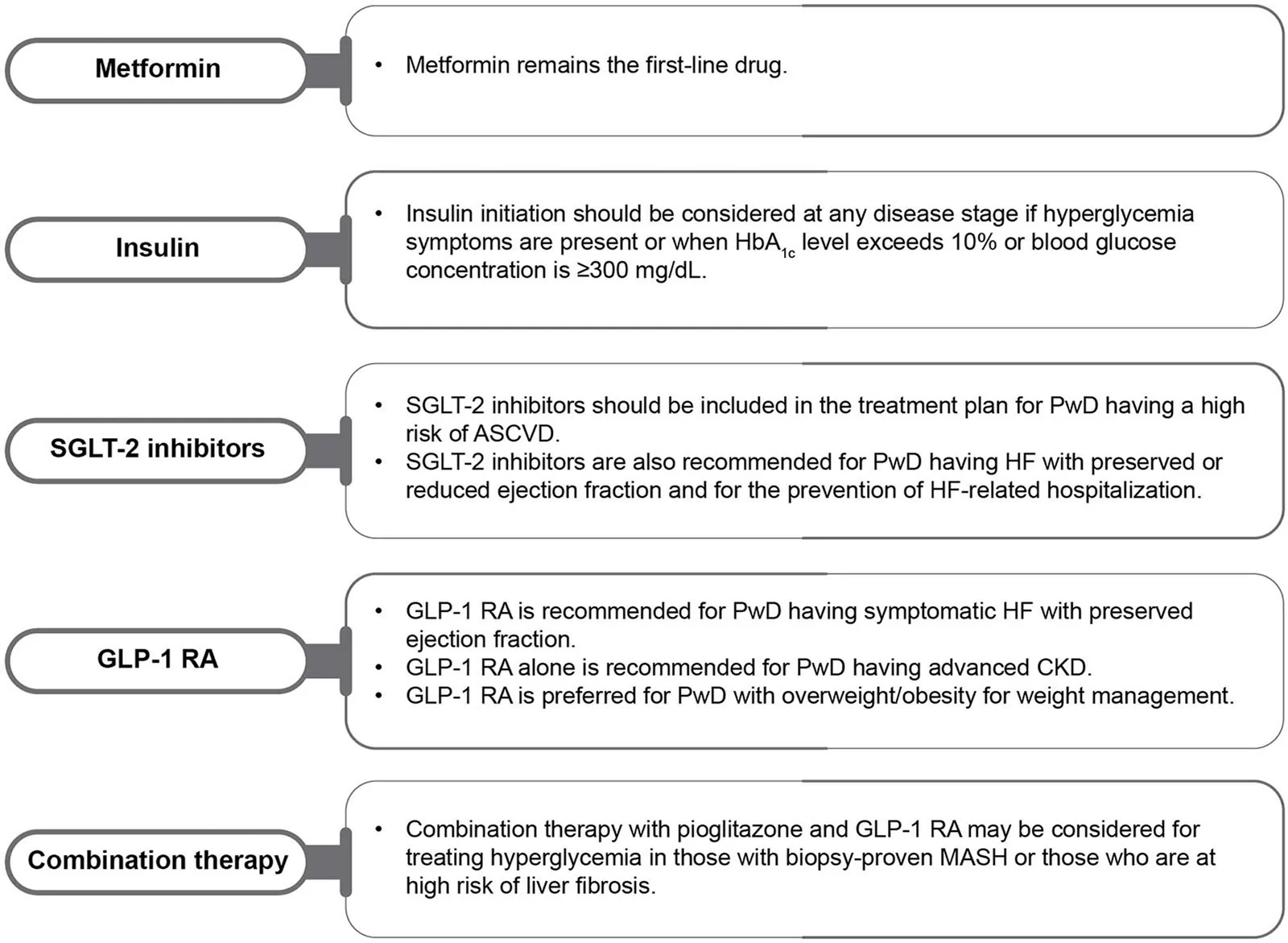
Choice of antihyperglycemic drugs for the management of diabetes ASCVD: Atherosclerotic cardiovascular disease; CKD: Chronic kidney disease; GLP-1 RA: Glucagon-like peptide-1 receptor agonist; HbA1c: Glycated hemoglobin; HF: Heart failure; MASH: Metabolic dysfunction-associated steatohepatitis; PwD: People with diabetes; SGLT-2: Sodium-glucose cotransporter-2 Figure conceptualized and created by the authors using Microsoft PowerPoint and Microsoft Word (Microsoft® Corp., Redmond, WA); the final artwork was professionally enhanced for publication.

To reduce cholesterol levels and prevent complications, moderate-intensity statin therapy is recommended for PwD aged 40-75 years without atherosclerotic cardiovascular disease (ASCVD); in PwD aged 20-39 years with additional ASCVD risk factors, initiation of statin therapy may be reasonable, and high-intensity statin therapy is recommended for PwD with high CVD risk [36]. Statin therapy is recommended to be continued in PwD aged over 75 years. However, in PwD with statin intolerance, bempedoic acid is recommended to reduce cardiovascular disease (CVD) events and lower cholesterol levels [36]. The opinions of the experts in the present study are in line with the ADA guidelines and emphasize considering gender and risk of complications, as well as affordability, acceptability, and accessibility of antidiabetic drugs for PwD.

Expert opinions: (1) Choice of antihypertensives: The experts emphasized that antihypertensive therapy should be individualized based on clinical indications, patient preferences, socioeconomic circumstances, and feasibility of monitoring. Angiotensin-converting enzyme inhibitors (ACEIs) or angiotensin receptor blockers (ARBs) were recommended as first-line agents for PwD with coexisting coronary artery disease or chronic kidney disease, provided appropriate monitoring can be ensured. When monitoring is challenging, alternative antihypertensive agents may be considered to achieve timely blood pressure control. The experts also highlighted the importance of balancing efficacy, affordability, and accessibility, with lifestyle modification and referral to affordable care options being important considerations in resource-limited settings.

(2) Choice of statins: The experts recommended tailoring statin therapy according to age, cardiovascular risk, comorbidities, treatment tolerance, and socioeconomic factors. They emphasized the use of the highest tolerated statin dose when treatment goals cannot be achieved with standard dosing and highlighted the importance of educating PwD regarding the benefits and potential AEs of statins. Shared decision-making was considered essential for improving adherence and achieving lipid targets.

(3) Choice of oral antidiabetic drugs (OADs): The experts agreed that metformin remains a preferred initial glucose-lowering agent for most PwD in the absence of contraindications. However, consistent with current guidelines, SGLT-2is and/or GLP-1 receptor agonists with proven cardiovascular or renal benefit are recommended for PwD with established ASCVD, heart failure, or chronic kidney disease, independent of metformin use or baseline HbA1c. Selection of additional glucose-lowering agents should be individualized based on comorbidities, treatment goals, patient preferences, and social determinants of health. SGLT-2 inhibitors and GLP-1 receptor agonists were recognized for their cardiovascular and renal benefits, particularly among individuals with established CVD, chronic kidney disease, or heart failure, although their accessibility and affordability remain challenging in resource-limited settings. Treatment selection should also consider body weight effects, severity of hyperglycemia, metabolic comorbidities, lifestyle factors, and the patient's overall clinical profile.

(4) Choice of body weight management: The experts highlighted that obesity management should follow a lifelong, person-centered approach that considers social, cultural, emotional, environmental, and economic factors. Patient engagement and shared decision-making were considered critical for successful weight management, particularly when selecting pharmacological or surgical interventions. The experts emphasized the use of person-first language, assessment of patient attitudes and readiness for change, provision of evidence-based information, and consideration of treatment affordability, accessibility, and long-term adherence. Effective weight management was viewed as requiring coordinated support from patients, HCPs, and healthcare systems.

Motivational Therapeutics

PwD often experience challenges in adopting the recommended behavioral changes necessary to prevent disease-related complications. Motivational interviewing (MI) is an evidence-based, empathetic counseling approach that supports patients by addressing and resolving ambivalence and enhancing motivation for change [37]. MI involves four processes: building a trusting relationship, focusing on a specific behavior to change, encouraging patients' motivation to change, and developing a plan to achieve the change [38].

The International Expert Panel Committee outlined key strategies for bringing about successful behavior change in PwD. These include establishing clear and simple communication between HCPs and PwD about the necessity of change, focusing on one recommendation at a time, and using the teach-back method to ensure understanding. HCPs should avoid the "one-size-fits-all" approach and should aim at tailored recommendations based on gender, age, available resources, and ethnicity of PwD [39]. The committee emphasized the importance of respecting patients' decisions, even when they differ from the HCP's recommendations. Proper training for PwD, regular follow-ups, and adjustments to treatment plans during each visit are also essential. Integrating planned behavior changes into daily routines, such as combining tooth brushing with blood glucose monitoring, can further support adherence. Finally, HCPs should adopt an empathetic and supportive tone, emphasizing encouragement rather than criticism or fear, to promote positive behavior change [39]. Meta-analyses reported that MI in PwD significantly reduced HbA1c, postprandial plasma glucose levels, systolic blood pressure, emotional distress, and depressive symptoms, while also enhancing self-efficacy [40,41]. More recent evidence from a 2025 meta-analysis further demonstrated improvements in HbA1c, fasting and two-hour postprandial glucose, self-management, and self-efficacy among adults with T2DM receiving transtheoretical model-based MI [42]. The experts in the present study also emphasized the effectiveness of MI and its implementation in the management of diabetes for a PCC approach, as summarized below.

Expert opinions: The experts recognized motivational therapy as a key component of PCC that promotes behavior change through encouragement, support, and patient engagement rather than prescriptive instructions. They emphasized that motivational strategies should be tailored to an individual's readiness for change, preferences, and circumstances to improve lifestyle modification and medication adherence. MI was identified as an important tool, while approaches such as the "adjacent possible" concept and the "3Is" framework (inform, incubate, and initiate) were considered useful for facilitating gradual and sustainable behavior change. The experts further noted that motivation should focus on practical, achievable goals and be adapted according to the patient's level of understanding, engagement, and relationship with the healthcare provider.

## Conclusions

Diabetes poses a multifaceted burden on the physical, emotional, and social well-being of PwD, affecting their overall QoL. Expert insights highlight the need for a culturally sensitive, PCC approach in Myanmar, despite barriers at the patient, HCP, system, and educational levels. Myanmar's cultural context, characterized by familial structure, strong Buddhist values, community support, and mindfulness practices, offers a unique opportunity to integrate PCC effectively. Implementing PCC stewardship through structured policies, motivational interviewing, and individualized treatment strategies, including antidiabetic agents and statins, can optimize glycemic control. Treatment decisions must consider age, comorbidities, cardiovascular risk, tolerability, and affordability to ensure patient-centered, evidence-based care.
